# Soluble Urokinase Receptor and Mortality in Kidney Transplant Recipients

**DOI:** 10.3389/ti.2021.10071

**Published:** 2022-02-03

**Authors:** Christian Morath, Salim S. Hayek, Bernd Döhler, Christian Nusshag, Claudia Sommerer, Martin Zeier, Jochen Reiser, Caner Süsal

**Affiliations:** ^1^ Department of Nephrology, Heidelberg University Hospital, Heidelberg, Germany; ^2^ Division of Cardiology, Department of Medicine, University of Michigan, Ann Arbor, MI, United States; ^3^ Institute of Immunology, Heidelberg University Hospital, Heidelberg, Germany; ^4^ Department of Medicine, Rush Medical College, Rush University, Chicago, IL, United States; ^5^ Transplant Immunology Research Center of Excellence, Koç Üniversitesi, Istanbul, Turkey

**Keywords:** mortality, kidney, transplantation, suPAR, cardiovascular

## Abstract

**Main problem:** Soluble urokinase plasminogen activator receptor (suPAR) is an immunological risk factor for kidney disease and a prognostic marker for cardiovascular events.

**Methods:** We measured serum suPAR levels in a total of 1,023 kidney transplant recipients either before (cohort 1, *n* = 474) or at year 1 after transplantation (cohort 2, *n* = 549). The association of suPAR levels and all-cause and cardiovascular mortality was evaluated by multivariable Cox regression analysis.

**Results:** The highest suPAR tertile compared to the two lower tertiles had a significantly higher risk of all-cause mortality in both cohorts separately (cohort 1: hazard ratio (HR) 1.92, 95% confidence interval (CI) 1.20–3.08, *p* = 0.007; cohort 2: HR = 2.78, 95% CI 1.51–5.13, *p* = 0.001) and combined (*n* = 1,023, combined HR = 2.14, 95% CI 1.48–3.08, *p* < 0.001). The association remained significant in the subgroup of patients with normal kidney function (cohort 2: HR = 5.40, 95% CI 1.42–20.5, *p* = 0.013). The increased mortality risk in patients with high suPAR levels was attributable mainly to an increased rate of cardiovascular death (*n* = 1,023, HR = 4.24, 95% CI 1.81–9.96, *p* < 0.001).

**Conclusion:** A high suPAR level prior to and at 1 year after kidney transplantation was associated with an increased risk of patient death independent of kidney function, predominantly from cardiovascular cause.

## Graphical Abstract

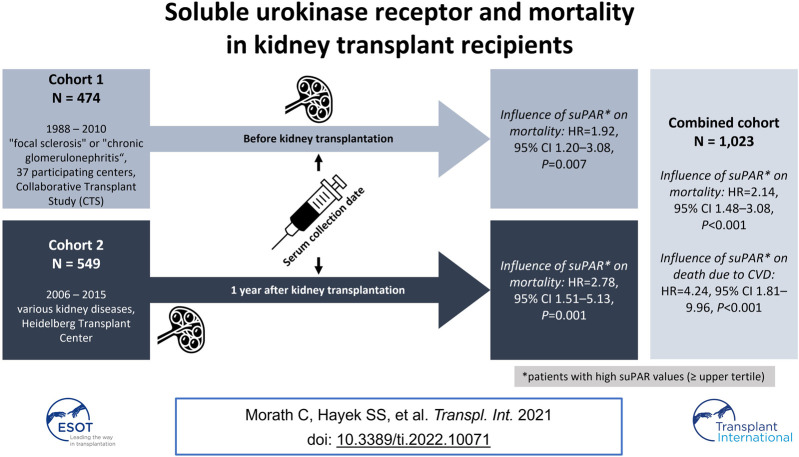

## Introduction

Graft survival after kidney transplantation is limited mainly for two reasons: first, chronic renal allograft dysfunction due to antibody-mediated rejection caused by development of *de novo* donor-specific antibodies and second, death with functioning allograft which in the long term is primarily caused by cardiovascular events (1,2). Early identification of patients at risk for cardiovascular events and cardiovascular death may not only reduce the mortality but also the number of graft losses caused by death of the patient with functioning graft.

The soluble urokinase plasminogen activator receptor (suPAR) is the soluble form of uPAR, the membrane-bound receptor for uPA (urokinase). suPAR is a risk factor for kidney disease, both acute and chronic and a biomarker for innate activation of the immune system (3–7). In several studies, serum levels of suPAR were reported to be associated with increased mortality in intensive care unit and septic patients (8,9). In addition, increased suPAR levels were found in patients with a high frequency of cardiovascular events and deaths in populations without chronic kidney disease (10–13). More recently, a high suPAR level was reported to be a predictor of total and cardiovascular mortality in 1,038 hemodialysis patients from 35 dialysis units in Italy (14).

To date, no major study with long-term follow-up has investigated the value of suPAR for the prediction of cardiovascular events and cardiovascular mortality in recipients of kidney transplants. We studied the association between suPAR measured before or at year 1 after transplantation and outcomes in a total of 1,023 kidney transplant recipients.

## Patients and Methods

### Study Population

SuPAR was measured pre-kidney transplant in cohort 1 consisting of 474 patients transplanted between 1988 and 2010 with the primary diagnosis of “focal sclerosis” or “chronic glomerulonephritis” from 37 participating centers that provided a pre-transplant serum from patients reported to the Collaborative Transplant Study (CTS, www.ctstransplant.org). SuPAR was measured 1 year post-kidney transplant in cohort 2, consisting of 549 patients aged 18 years or older who were transplanted at the Heidelberg Transplant Center from 2006 to 2015. The primary diagnosis at the time of transplant was autoimmune disease in 3.1%, disease of blood and blood forming organs in 0.9%, congenital disease in 4.4%, polycystic disease in 16.2%, diabetes in 10.0%, chronic glomerulonephritis in 26.6%, IgA nephropathy in 12.9%, interstitial nephritis in 10.2%, metabolic disease in 2.7%, vascular disease in 4.6%, and other diseases in 1.1% of the patients. In 7.3% of the cases, the original disease could not be specified.

### Ethics

The work of the CTS is approved by the Ethics Committee of the Medical Faculty of Heidelberg University (No. 083/2005) and performed in accordance with the World Medical Association Declaration of Helsinki Ethical Principles in the currently valid version.

### suPAR Measurements

Serum suPAR was measured in a blinded fashion using either the uPAR Quantikine® ELISA kit (R&D, Minneapolis, MN, United States; cohort 1) or the suPARnostic kit (ViroGates, Birkerød, Denmark; cohort 2) according to the manufacturer’s instructions. The lower detection limit is less than 33 and 100 pg/ml, the intra-assay variation less than 5 and 2.75%, and the inter-assay variation less than 5 and 9.17% for the uPAR Quantikine® ELISA and suPARnostic kit, respectively.

### Outcomes

The information on date and cause of patient’s death was derived from CTS basic follow up forms that are filled out by participating centers at post-transplant months 3, 6, 12 and yearly thereafter.

### Statistics

Time to death was calculated from the serum collection date (cohort 1: serum collection date = transplant date, cohort 2: serum collection date = 1 year after transplantation). Multivariable Cox regression analysis was performed to account for the possible influence of the following confounders separately according to cohort: cohort number, transplant year, transplant number, recipient and donor age, recipient and donor sex, donor relationship, pre-transplant human leukocyte antigen (HLA) antibodies, cold ischemia time (deceased donor), time on dialysis, HLA A + B + DR mismatches, and existence of comorbidities (pretransplant cancer, diabetes mellitus, other reasons of moderate or poor evaluation of the patient as candidate for transplantation). Analysis in cohort 2 included the following additional variables which were not available in cohort 1: rejection treatment during first post-transplant year, 1-year serum creatinine, immunosuppressive therapy at year 1, and presence of an increased cardiovascular risk at year 1 (diabetes mellitus, hypertension, smoking, hypercholesterolemia, obesity). Survival rates were illustrated using the Kaplan-Meier method. The software package IBM SPSS Statistics (SPSS Inc., Chicago, IL, United States) was used.

## Results

### Patient Demographics

SuPAR levels were measured in a total of 1,023 kidney transplant recipients either before (cohort 1, *n* = 474) or at year 1 after transplantation (cohort 2, *n* = 549). In addition to the time of serum sampling, the two cohorts differed with respect to the year of transplantation (cohort 1: 1988–2010, cohort 2: 2006–2015), the geographical region (cohort 1: multicenter, multinational CTS Serum Study, cohort 2: single-center study, Heidelberg, Germany), the donor relationship, the recipient sex, and the donor and recipient age (Table 1, Supplementary Tables 1, 2). In cohort 1, all patients had marginal renal function at the time of suPAR measurement and suPAR levels did not significantly differ between patients with tissue diagnosis “focal sclerosis” and “chronic glomerulonephritis” (median 5.7 versus 5.8 ng/ml, *p* = 0.51). In cohort 2, 46.4% of patients had a serum creatinine of <130 μmol/L, 49.0% of patients a serum creatinine of 130–260 μmol/L, and 4.6% of patients a serum creatinine of >260 μmol/L at the time of suPAR measurement at year one.

**TABLE 1:** Demographics of study patients, *n* (%).

Characteristic	Unknown (%)	Cohort 1 *n* = 474	Cohort 2 *n* = 549	*p*
Geographical region	–			–
Europe		242 (51%)	549 (100%)	
North America		168 (35%)	–	
Other		64 (14%)	–	
Transplant year	–			–
Range		1988–2010	2006–2015	
Median		2003	2011	
Transplant number	–			0.12
First transplant		402 (85%)	484 (88%)	
Retransplant		72 (15%)	65 (12%)	
Donor relationship	–			<0.001
Living		120 (25%)	217 (40%)	
Deceased		354 (75%)	332 (60%)	
Recipient sex	–			0.001
Female		155 (33%)	233 (42%)	
Male		319 (67%)	316 (58%)	
Recipient age (years)	–			<0.001
<18		52 (11%)	–	
18–59		342 (72%)	403 (73%)	
≥60		80 (17%)	146 (27%)	
Mean ± SD		41.8 ± 17.0	48.4 ± 14.1	<0.001
Donor age (years)	0.3			<0.001
<18		18 (8%)	13 (2%)	
18–59		364 (77%)	360 (66%)	
≥60		69 (15%)	176 (32%)	
Mean ± SD		42.2 ± 16.1	52.2 ± 14.8	<0.001
suPAR (ng/ml)	–			
Range		1.0–26.4	1.1–18.1	
Median (Tertiles)		5.7 (4.9; 6.7)	6.2 (5.2; 7.2)	

SD, standard deviation.

### Serum suPAR Levels and Mortality

**FIGURE 1:**
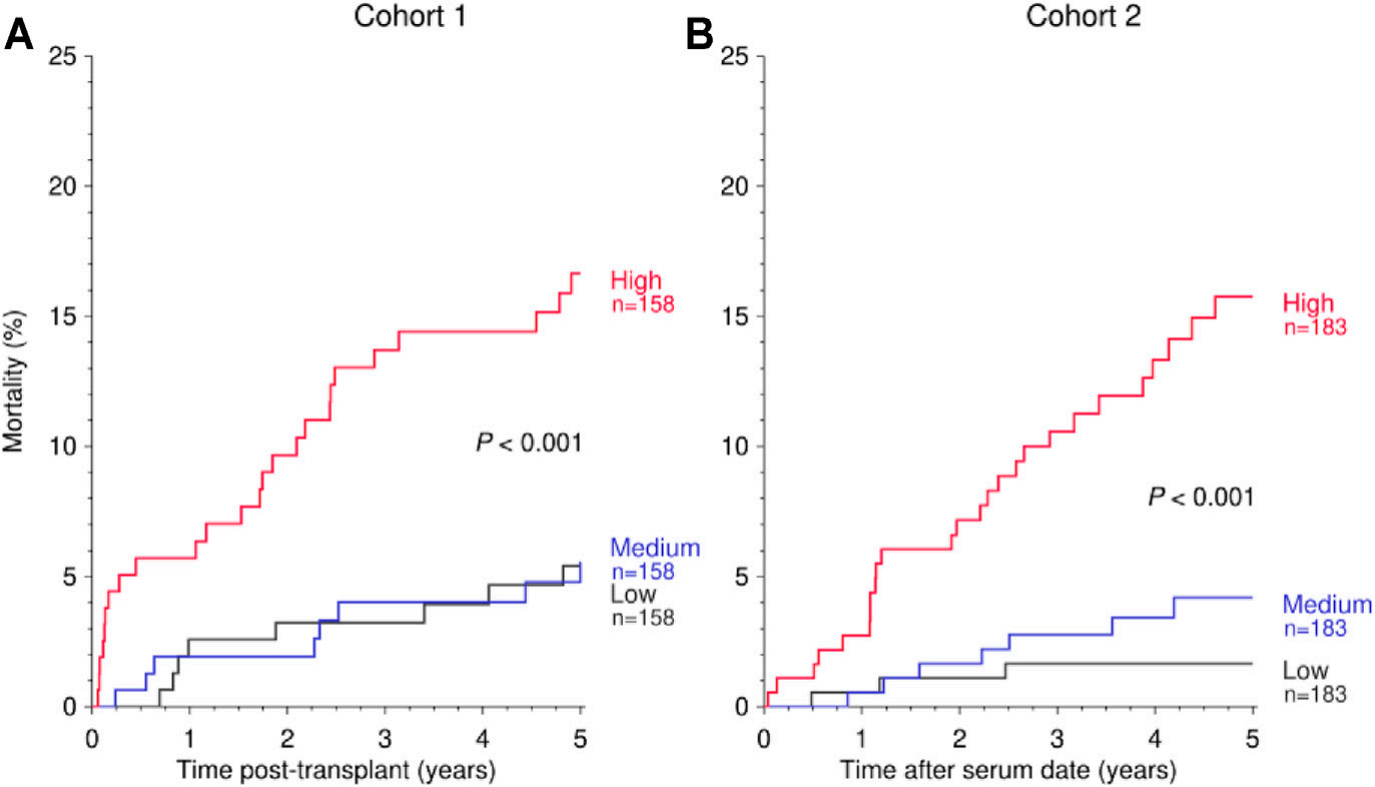
Kaplan-Meier curves demonstrating the impact of suPAR (ng/ml) on 5-year mortality post-transplant in cohort 1 **(A)** and after serum collection date in cohort 2 **(B)**. The categories “Low,” “Medium,” and “High” are defined by the tertiles of suPAR in each cohort. Log rank *p* values for trend are shown.

The risk of mortality was significantly higher in patients in the high than in the medium or low tertiles (“normal”) of suPAR levels (cohort 1: hazard ratio (HR) 1.92, 95% confidence interval (CI) 1.20–3.08, *p* = 0.007; cohort 2: HR = 2.78, 95% CI 1.51–5.13, *p* = 0.001; Figure 1 and Table 2). To exclude a decisive influence of kidney function on baseline suPAR levels and subsequent outcomes in cohort 2, we analyzed the impact on mortality of suPAR in patients with good kidney function, e.g. a serum creatinine of <130 μmol/L, separately. Also in this subgroup, the mortality risk was significantly higher in patients with high compared to normal suPAR levels (cohort 2 with good kidney function: HR = 5.40, 95% CI 1.42–20.5, *p* = 0.013; Table 2).

**TABLE 2:** Results of the multivariable Cox regression analysis for influence of suPAR on mortality after serum collection date.

Subpopulation	N	HR	95% CI	*p*-value
All study patients	1,023	2.14	1.48–3.08	**<0.001**
Death due to CVD		4.24	1.81–9.96	**<0.001**
Death due to infection		2.20	0.90–5.39	0.083
Death due to cancer		1.61	0.53–4.91	0.40
Cohort 1	474	1.92	1.20–3.08	**0.007**
Cohort 2	549	2.78	1.51–5.13	**0.001**
Good kidney function	255	5.40	1.42–20.5	**0.013**
Female patients	388	1.91	0.97–3.76	0.061
Male patients	635	2.41	1.56–3.73	**<0.001**
Young patients <50 years	584	3.38	1.81–6.34	**<0.001**
Elderly patients ≥50 years	439	1.73	1.10–2.71	**0.017**

Hazard ratios (HR) with 95% confidence interval (CI) of patients with high suPAR values (≥upper tertile) are shown. Significant *p-*values marked bold.

**FIGURE 2:**
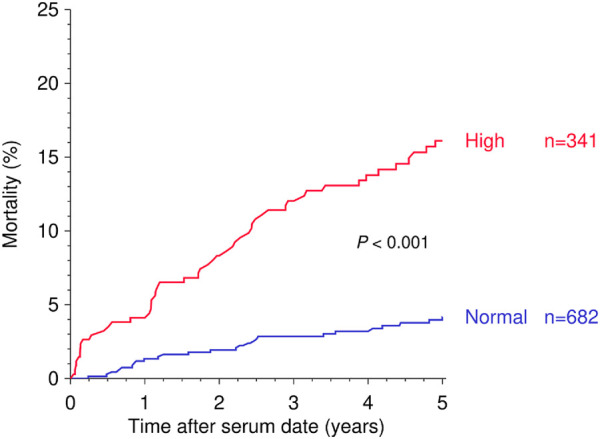
Kaplan-Meier curves demonstrating the impact of suPAR (ng/ml) above the upper tertile (“High”) against suPAR values below the upper tertile (“Normal”) on 5-year mortality after serum collection date. Log rank *p* value is shown.

**FIGURE 3:**
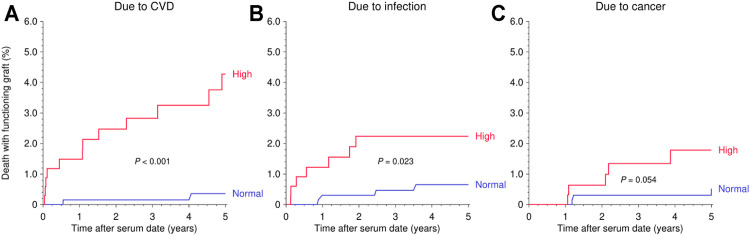
Kaplan-Meier curves demonstrating the impact of suPAR (ng/ml) on death with a functioning graft in the following 5 years after serum collection date as stratified by cause of death. Log rank *p* value is shown. **(A)** Due to CVD. **(B)** Due to infection. **(C)** Due to cancer.

**FIGURE 4:**
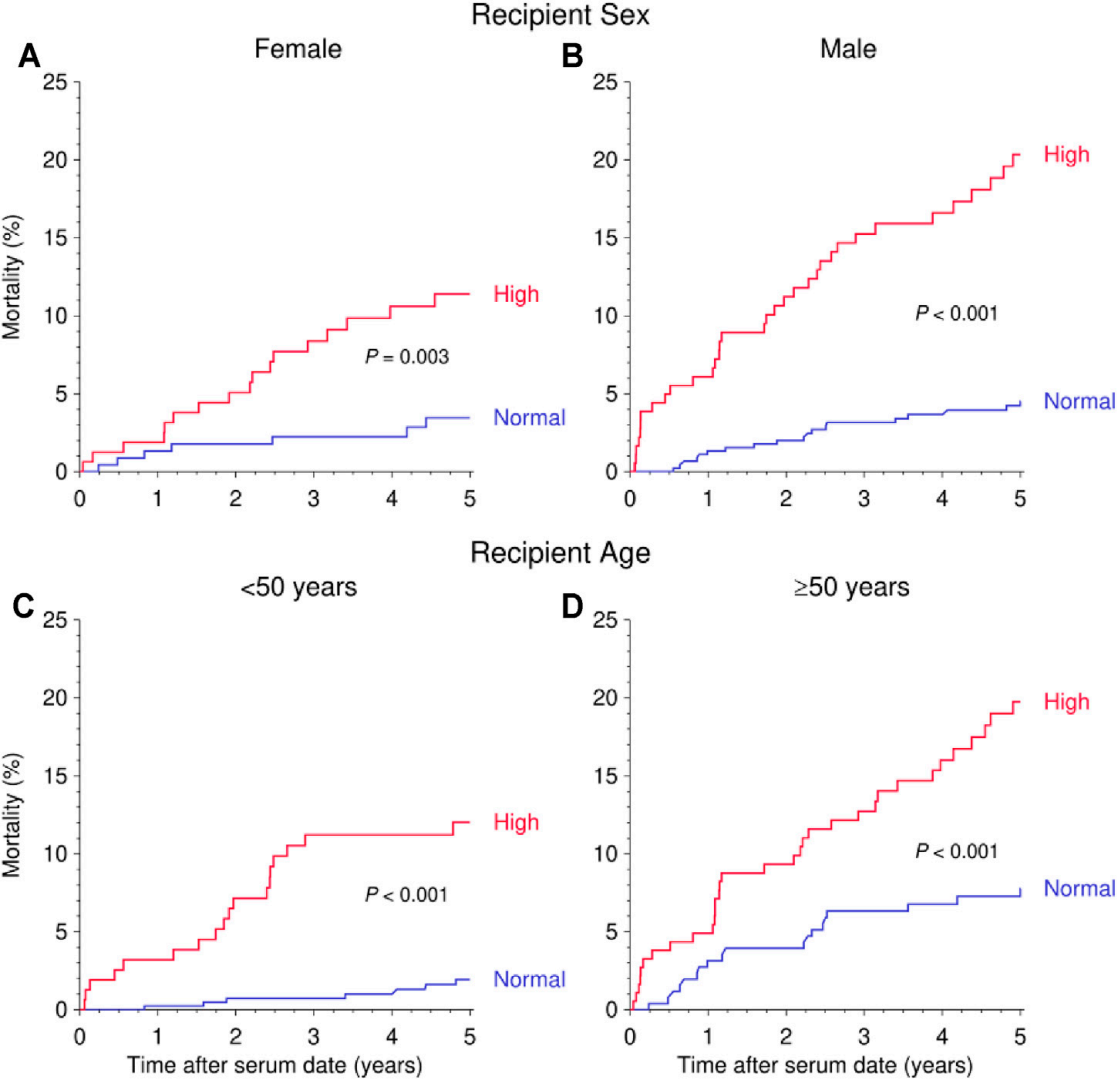
Kaplan-Meier curves demonstrating the impact of suPAR (ng/ml) on 5-years mortality after serum collection date as stratified by recipient sex **(A,B)** and recipient age **(C,D)**. Log rank *p* value is shown.

Due to the rather comparable risk and a similar distribution of suPAR levels in cohorts 1 and 2 (Supplementary Figure 1), both cohorts were combined for further in-depth analysis. The combined risk of mortality during follow-up after the pre- or post-transplant measurement was more than 2-fold higher in patients with a high than normal suPAR level (HR = 2.14, 95% CI 1.48–3.08, *p* < 0.001; Figure 2 and Table 2). As illustrated in Figure 3 and Table 2, the mortality in patients with a high suPAR level was attributable rather to cardiovascular death with a striking HR of 4.24 (95% CI 1.81–9.96, *p* < 0.001) than to death from infection or cancer (infection: HR = 2.20, 95% CI 0.90–5.39, *p* = 0.083; cancer: HR = 1.61, 95% CI 0.53–4.91, *p* = 0.40). The impact of high suPAR level on all-cause mortality was more pronounced in male patients (HR = 2.41, 95% CI 1.56–3.73, *p* < 0.001) than in female patients (HR = 1.91, 95% CI 0.97–3.76, *p* = 0.061; Table 2 and Figures 4A,B) and in younger patients aged <50 years (HR = 3.38, 95% CI 1.81–6.34, *p* < 0.001) than in ≥50-year-old patients (HR = 1.73, 95% CI 1.10–2.71, *p* = 0.017; Table 2 and Figures 4C,D).

## Discussion

In this study of 1,023 patients, serum suPAR level was a robust predictor of all-cause mortality after kidney transplantation. A high compared to normal suPAR level was associated with more than doubled risk of mortality during follow-up. This finding was consistent and independent of the time of transplantation (cohort 1: 1988–2010, cohort 2: 2006–2015), the primary kidney disease (cohort 1: glomerulonephritis, cohort 2: various), the time of serum sampling (cohort 1: before transplantation, cohort 2: 1 year after transplantation), or the suPAR assay used (cohort 1: uPAR Quantikine^®^ ELISA kit, cohort 2: suPARnostic kit). The findings were confirmed independently in female versus male, or elderly versus young patients, and most importantly, the influence of suPAR on mortality was constant in patients with different levels of kidney function (cohort 1: marginal kidney function; cohort 2: different levels of kidney function; subgroup of cohort 2 with good kidney function at year 1 and a serum creatinine <130 μmol/L). The main cause of mortality was cardiovascular death with a striking HR of 4.24 in patients with high suPAR level.

SuPAR had been implicated as a biomarker for cardiovascular events and cardiovascular death in the general population as well as in patients with specific diseases, such as type 1 diabetes mellitus and coronary artery disease, or in patients undergoing coronary angiography (11,12,15–17). SuPAR predicted all-cause and cardiovascular mortality independent of classical risk factors or cardiac biomarkers, such as NT-pro BNP, or inflammatory markers, such as CRP. In different studies, suPAR was a strong predictor of cardiovascular death, even after adjustment for cardiovascular risk factors or kidney function (11,12). In kidney disease, suPAR acts as both, a biomarker for future kidney disease as well as being causally implicated through podocyte integrin signaling and tubular cell mitochondrial metabolic adaptation (3,4,18). However, evidence that support the direct involvement of suPAR in cardiovascular mortality is limited and the reported strong associations require to be followed up with translational studies that address the question of suPAR being a cause of cardiovascular disease. Until then, it remains unclear whether there is a causal relationship between elevated suPAR levels and a higher risk of disease or death or whether elevated suPAR levels are merely an unmodifiable marker of disease progression.

Several points are particularly noteworthy in our study. First, regardless of the suPAR assay used, uPAR Quantikine^®^ ELISA kit or suPARnostic kit, the serum suPAR levels in our study were with a median of 5.7 in cohort 1 and 6.2 ng/ml in cohort 2 higher than the median levels reported in other cardiovascular mortality studies with, i.e., a median suPAR level of 3.0 ng/ml in the study by Sommerer et al. (11). The high median level measured in our study can be explained in part by the impaired renal function in kidney transplant recipients (cohort 2) and especially patients awaiting kidney transplantation (cohort 1) due to accumulation of suPAR as result of decreased renal excretion (4). However, the higher suPAR levels in our study may also be an indicator of increased cardiovascular comorbidity of patients with chronic kidney disease. These assumptions are supported by a recently published study from Italy on the predictive value of suPAR on all-cause and cardiovascular mortality in hemodialysis patients (14). In this study, the median suPAR level was with 6.25 ng/ml even slightly higher than that found in the two cohorts studied by us. Second, in patients with high suPAR levels an impressively increased risk of mortality with a HR of 2.14 was observed. In particular the mortality due to cardiovascular death was with a HR of 4.24 above the figures published in other cohorts with, i.e., a median HR for cardiovascular death of 3.43 for the highest suPAR quartile in the study by Sommerer et al. and a HR of 1.48 in Italian dialysis patients when a comparable cut-off as in our study was used (11,14). This in turn could indicate the high susceptibility to cardiovascular death of patients with chronic kidney disease on the waiting list or after transplantation, with high suPAR levels being a strong predictor of increased mortality. Third, high suPAR levels predicted risk of mortality independent of kidney function. The HR for mortality was 1.92 (*p* = 0.007) in patients with marginal kidney function awaiting transplantation (cohort 1), 2.78 (*p* = 0.001) in patients with different levels of kidney function 1 year after transplantation (cohort 2), and 5.40 (*p* = 0.013) when only the subgroup of patients with a serum creatinine <130 μmol/L was analyzed (cohort 2 with good kidney function). This suggests that the link between suPAR and increased mortality is not related to a decreased kidney function, but rather to an unrelated process, i.e., chronic inflammation.

Among the influential factors that were considered in the multivariable analysis, recipient age, suPAR level, and transplant year had the strongest impact on mortality. Pre-transplant cancer, diabetes mellitus, and other reasons of moderate or poor evaluation of the patient as candidate for transplantation (for cohorts 1 and 2), and presence of an increased cardiovascular risk at year 1 (for cohort 2 only) were also considered; however, none of them showed a significant influence. Limitations of the current study include the selection of different cohorts of patients with different underlying diseases (“focal sclerosis” or “chronic glomerulonephritis” in cohort 1 versus various kidney diseases in cohort 2), different timing of serum sample collection (pre-transplant in cohort 1 versus 1 year post-transplant in cohort 2), and the use of different assays for suPAR measurements (uPAR Quantikine^®^ ELISA kit in cohort 1 versus suPARnostic kit in cohort 2). However, as suPAR levels were distributed similarly and not normally in both cohorts and the outcome was similar when cohorts 1 and 2 were analyzed separately, we felt that it was justified to combine both cohorts for further in depth analysis. Moreover, that suPAR levels predicted inferior outcome independent of the primary kidney disease, the time of serum sampling, or the assay, especially in the highest tertile of patients, can also be interpreted as a strength that underlines the robustness of our findings.

In conclusion, a high serum suPAR level was found to be a strong and robust predictor of all-cause and cardiovascular mortality in kidney transplant recipients that may allow for better risk stratification and early intervention in high-risk patients. Most importantly, prediction of risk by suPAR was independent of kidney function at baseline.

## Capsule Summary Sentence

The soluble urokinase plasminogen activator receptor (suPAR) is a risk factor for cardiovascular disease and cardiovascular death in chronic kidney disease and non-chronic kidney disease populations. In hemodialysis patients, the risk of death was almost two times higher in patients with suPAR levels in the highest compared to the lowest tertile with a significantly increased risk of cardiovascular death. So far, no study examined the association of high suPAR levels with overall and cardiovascular mortality in kidney transplant recipients. In two independent cohorts with a total of 1,023 kidney transplant recipients a serum suPAR level in the highest compared to the two lower tertiles was a strong predictor of death, predominantly from cardiovascular cause. These findings were confirmed in different subcohorts. Most importantly, prediction of overall and cardiovascular death was independent of the baseline kidney function indicating that mortality may not be related to a decreased kidney function, but rather to an unrelated process such as chronic inflammation. SuPAR may help to identify kidney transplant recipients at a high risk of cardiovascular death and enable to provide them with the best follow-up and post-transplant care.

## Data Availability

The raw data are available upon request to the Collaborative Transplant Study in accordance with the consents of the patients and the participating transplant centers and registries.

## Abbreviations

CI, confidence interval; CTS, Collaborative Transplant Study; HR, hazard ratio; HLA, human leukocyte antigen; suPAR, soluble urokinase plasminogen activator receptor; uPA, urokinase.
