# Supplementary material for: Soluble Urokinase Receptor and Mortality in Kidney Transplant Recipients
Source: Transpl Int. 2022 Feb 3;35:10071. doi: 10.3389/ti.2021.10071 (PMC8842271; doi:10.3389/ti.2021.10071)
Supplement: Supplementary file 1 [file Table1.docx]

**Supplemental Table 1.** Demographics of cohort 1 stratified by tertiles of suPAR, n (%)

| **Characteristic** | **Unknown (%)** | **Low n=158** | **Medium n=158** | **High n=155** | ***P*** |
| --- | --- | --- | --- | --- | --- |
| Geographical region  Europe  North America  Other | – | 76 (48%) 56 (35%) 26 (16%) | 76 (48%) 65 (41%) 17 (11%) | 90 (57%) 47 (30%) 21 (13%) | 0.13 |
| Transplant year  Median  IQR | – | 2003 2001–2006 | 2004 2001–2006 | 2004 2001–2007 | 0.12 |
| Transplant number  First transplant  Retransplant | – | 140 (89%) 18 (11%) | 133 (84%) 25 (16%) | 129 (82%) 29 (18%) | 0.085 |
| Donor relationship  Living  Deceased | – | 58 (37%) 100 (63%) | 40 (25%) 118 (75%) | 22 (14%) 136 (86%) | <0.001 |
| Recipient sex  Female  Male | – | 38 (24%) 120 (76%) | 41 (26%) 117 (74%) | 76 (48%) 82 (52%) | <0.001 |
| Recipient age (years)  <18  18–59  ≥60  Mean±SD | – | 21 (13%) 124 (78%) 13 (  8%)  37.3±15.8 | 11 (  7%) 117 (74%) 30 (19%)  43.1±16.0 | 20 (13%) 101 (64%) 37 (23%)  44.9±18.4 | 0.007  <0.001 |
| Donor age (years)  <18  18–59  ≥60  Mean±SD | 0.6 | 11 (  7%) 125 (79%) 22 (14%)  41.5±15.7 | 12 (  8%) 119 (75%) 27 (17%)  43.0±15.8 | 15 (10%) 120 (77%) 20 (13%)  42.2±17.0 | 0.49  0.57 |

SD, standard deviation; IQR, interquartile range
