# Supplementary material for: Soluble Urokinase Receptor and Mortality in Kidney Transplant Recipients
Source: Transpl Int. 2022 Feb 3;35:10071. doi: 10.3389/ti.2021.10071 (PMC8842271; doi:10.3389/ti.2021.10071)
Supplement: Supplementary file 2 [file Table2.docx]

**Supplemental Table 2.** Demographics of cohort 2 stratified by tertiles of suPAR, n (%)

| **Characteristic** | **Unknown (%)** | **Low n=183** | **Medium n=183** | **High n=183** | ***P*** |
| --- | --- | --- | --- | --- | --- |
| Transplant year  Median  IQR | – | 2011 2009–2013 | 2011 2009–2012 | 2010 2009–2012 | 0.015 |
| Transplant number  First transplant  Retransplant | – | 165 (90%) 18 (10%) | 161 (88%) 22 (12%) | 158 (86%) 25 (14%) | 0.26 |
| Donor relationship  Living  Deceased | – | 99 (54%) 84 (46%) | 68 (37%) 115 (63%) | 50 (27%) 133 (73%) | <0.001 |
| Recipient sex  Female  Male | – | 74 (40%) 109 (60%) | 75 (41%) 108 (59%) | 84 (46%) 99 (54%) | 0.29 |
| Recipient age (years)  18–59  ≥60  Mean±SD | – | 161 (88%) 22 (12%)  44.0±13.2 | 135 (74%) 48 (26%)  49.1±13.6 | 107 (58%) 76 (42%)  52.0±14.3 | <0.001  <0.001 |
| Donor age (years)  <18  18–59  ≥60  Mean±SD | – | 1 (  1%) 137 (75%) 45 (25%)  49.6±13.2 | 8 (  4%) 122 (67%) 53 (29%)  51.0±16.1 | 4 (  2%) 101 (55%) 78 (43%)  56.0±14.4 | 0.002  <0.001 |

SD, standard deviation; IQR, interquartile range
